# Rapid identification of pathogenic bacteria using Raman spectroscopy and deep learning

**DOI:** 10.1038/s41467-019-12898-9

**Published:** 2019-10-30

**Authors:** Chi-Sing Ho, Neal Jean, Catherine A. Hogan, Lena Blackmon, Stefanie S. Jeffrey, Mark Holodniy, Niaz Banaei, Amr A. E. Saleh, Stefano Ermon, Jennifer Dionne

**Affiliations:** 1 Dept. of Applied Physics, Stanford University, Stanford, CA USA; 2 Dept. of Materials Science and Engineering, Stanford University, Stanford, CA USA; 3 Dept. of Computer Science, Stanford University, Stanford, CA USA; 4 Dept. of Electrical Engineering, Stanford University, Stanford, CA USA; 5 Dept. of Pathology, Stanford University School of Medicine, Stanford, CA USA; 6 Clinical Microbiology Laboratory, Stanford Health Care, Stanford, CA USA; 7 Dept. of Surgery, Stanford University School of Medicine, Stanford, CA USA; 8 Dept. of Medicine, Stanford University School of Medicine, Stanford, CA USA; 9 VA Palo Alto Health Care System, Palo Alto, CA USA; 10 Division of Infectious Diseases and Geographic Medicine, Department of Medicine, Stanford University School of Medicine, Stanford, CA USA; 11 Dept. of Engineering Mathematics and Physics, Faculty of Engineering, Cairo University, Giza, Egypt

**Keywords:** Machine learning, Clinical microbiology, Pathogens, Raman spectroscopy

## Abstract

Raman optical spectroscopy promises label-free bacterial detection, identification, and antibiotic susceptibility testing in a single step. However, achieving clinically relevant speeds and accuracies remains challenging due to weak Raman signal from bacterial cells and numerous bacterial species and phenotypes. Here we generate an extensive dataset of bacterial Raman spectra and apply deep learning approaches to accurately identify 30 common bacterial pathogens. Even on low signal-to-noise spectra, we achieve average isolate-level accuracies exceeding 82% and antibiotic treatment identification accuracies of 97.0±0.3%. We also show that this approach distinguishes between methicillin-resistant and -susceptible isolates of *Staphylococcus aureus* (MRSA and MSSA) with 89±0.1% accuracy. We validate our results on clinical isolates from 50 patients. Using just 10 bacterial spectra from each patient isolate, we achieve treatment identification accuracies of 99.7%. Our approach has potential for culture-free pathogen identification and antibiotic susceptibility testing, and could be readily extended for diagnostics on blood, urine, and sputum.

## Abstract (web summary)

The use of Raman spectroscopy for pathogen identification is hampered by the weak Raman signal and phenotypic diversity of bacterial cells. Here the authors generate an extensive dataset of bacterial Raman spectra and apply deep learning to identify common bacterial pathogens and predict antibiotic treatment from noisy Raman spectra.

## Introduction

Bacterial infections are a leading cause of death in both developed and developing nations, taking >6.7 million lives each year^1,2^. These infections are also costly to treat, accounting for 8.7% of annual healthcare spending, or $33 billion, in the United States alone^3^. Current diagnostic methods require sample culturing to detect and identify the bacteria and its antibiotic susceptibility, a slow process that can take days even in state-of-the-art labs^4,5^. Broad spectrum antibiotics are often prescribed while waiting for culture results^6^, and according to the Centers for Disease Control and Prevention, over 30% of patients are treated unnecessarily^7^. New methods for rapid, culture-free diagnosis of bacterial infections are needed to enable earlier prescription of targeted antibiotics and help mitigate antimicrobial resistance.

**Fig. 1:**
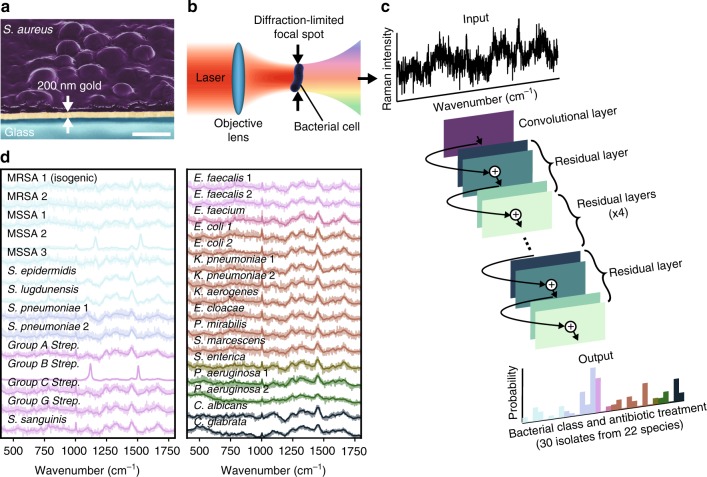
A convolutional neural network (CNN) can be used to identify bacteria from Raman spectra. **a** To build a training dataset of Raman spectra, we deposit bacterial cells onto gold-coated silica substrates and collect spectra from 2000 bacteria over monolayer regions for each strain. An SEM cross section of the sample is shown (gold coated to allow for visualization of bacteria under electron beam illumination). Scale bar is 1 µm. **b** Conceptual measurement schematic: by focusing the excitation laser source to a diffraction-limited spot size, Raman signal from single cells can be acquired. **c** Using a one-dimensional residual network with 25 total convolutional layers (see Methods for details), low-signal Raman spectra are classified as one of 30 isolates, which are then grouped by empiric antibiotic treatment. **d** Raman spectra of bacterial species can be difficult to distinguish, and short integration times (1 s) lead to noisy spectra (SNR = 4.1). Averages of 2000 spectra from 30 isolates are shown in bold and overlaid on representative examples of noisy single spectra for each isolate. Spectra are color-grouped according to antibiotic treatment. These reference isolates represent over 94% of the most common infections seen at Stanford Hospital in the years 2016–17^39^

Raman spectroscopy has the potential to identify the species and antibiotic resistance of bacteria, and when combined with confocal spectroscopy, can interrogate individual bacterial cells (Fig. 1a, b). Different bacterial phenotypes are characterized by unique molecular compositions, leading to subtle differences in their corresponding Raman spectra. However, because Raman scattering efficiency is low (~10^−8^ scattering probability^8^), these subtle spectral differences are easily masked by background noise. High signal-to-noise ratios (SNRs) are thus needed to reach high identification accuracies^9^, typically requiring long measurement times that prohibit high-throughput single-cell techniques. Additionally, the large number of clinically relevant species, strains, and antibiotic resistance patterns require comprehensive datasets that are not gathered in studies that focus on differentiating between species^10,11^, isolates (typically referred to as strains in the literature)^12,13^, or antibiotic susceptibilities^14–19^. In this work, we address this challenge by training a convolutional neural network (CNN) to classify noisy bacterial spectra by isolate, empiric treatment, and antibiotic resistance.

## Results

### Deep learning for bacterial classification from Raman spectra

In order to gather a training dataset, we measure Raman spectra using short measurement times on dried monolayer samples, as illustrated in Fig. 1. We ensure that the majority of individual spectra are taken over single cells and preparation conditions are consistent between samples (See Methods). We construct reference datasets of 60,000 spectra from 30 bacterial and yeast isolates for 3 measurement times — these 30 isolate classes cover over 94% of all bacterial infections treated at Stanford Hospital in the years 2016–17 and are representative of the majority of infections in intensive care units worldwide^20^. We further augment our reference dataset with 12,000 spectra from clinical patient isolates, including MRSA and MSSA isolates (see Methods for full dataset information). Previously, the lack of large datasets prohibited the use of CNNs due to the high number of spectra per bacterial class needed for training.

In recent years, CNNs have been applied with tremendous success to a broad range of computer vision problems^21–30^. However, while classical machine learning techniques have been applied to spectral data^11,12,14,31,32^, relatively little work has been done in adapting deep learning models to spectral data^33–36^. In particular, state-of-the-art CNN techniques from image classification such as residual connections have previously not been applied to low SNR, 1D spectral data. Our CNN architecture consists of 25 1D convolutional layers and residual connections^37^ — instead of two-dimensional images, it takes one-dimensional spectra as input (see Methods for further detail). Unlike previous work, we do not use pooling layers and instead use strided convolutions with the goal of preserving the exact locations of spectral peaks^38^. Empirically, we find that this strategy improves model performance.

We train the neural network on a 30-class isolate identification task, where the CNN outputs a probability distribution across the 30 reference isolates and the maximum is taken as the predicted class. The model is trained on the reference dataset and tested on an independent test dataset gathered from separately cultured samples.

**Fig. 2:**
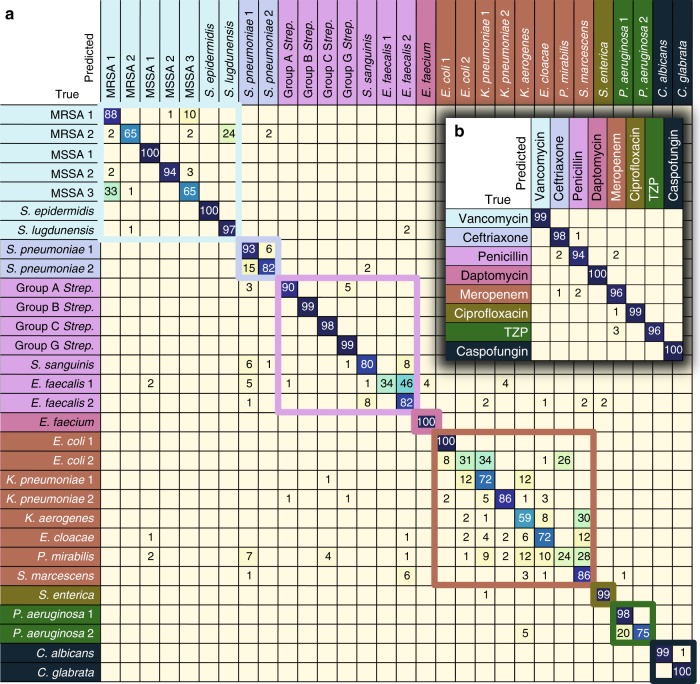
CNN performance breakdown by class. The trained CNN classifies 30 bacterial and yeast isolates with isolate-level accuracy of 82.2±0.3% and antibiotic grouping-level accuracy of 97.0±0.3% (± calculated as standard deviation across 5 train and validation splits). **a** Confusion matrix for 30 strain classes. Entry i, j represents the percentage out of 100 test spectra that are predicted by the CNN as class j given a ground truth of class i; entries along the diagonal represent the accuracies for each class. Misclassifications are mostly within antibiotic groupings, indicated by colored boxes, and thus do not affect the treatment outcome. Values below 0.5% are not shown, and matrix entries covered by figure insets are all below 0.5% aside from a 2% misclassification of MRSA 2 as *P. aeruginosa* 1 and 1% misclassification of Group B *Strep*. as *K. aerogenes*. **b** Predictions can be combined into antibiotic groupings to estimate treatment accuracy. TZP = piperacillin-tazobactam. All values below 0.5% are not shown

A performance breakdown for individual classes is displayed in the confusion matrix in Fig. 2a. Here, we show data for 1 s measurement times, corresponding to a SNR of 4.1 — roughly an order of magnitude lower than typical reported bacterial spectra^10–12^; classification accuracies increase with SNR, as shown in Supplementary Fig. 1. On the 30-class task, the average isolate-level accuracy is 82.2±0.3% (± calculated as standard deviation across 5 train and validation splits). Gram-negative bacteria are primarily misclassified as other Gram-negative bacteria; the same is generally true for Gram-positive bacteria, where additionally, the majority of misclassifications occur within the same genus. In comparison, our implementations of the more common classification techniques of logistic regression and support vector machine (SVM) achieve accuracies of 75.7% and 74.9%, respectively.

### Identification of empiric treatments and antibiotic resistance

Species-level classification accuracy is the standard metric for bacterial identification, but in practice, the priority for physicians is choosing the correct antibiotic to treat a patient. Common antibiotics often have activity against multiple species, so the 30 isolates can be arranged into groupings based on the recommended empiric treatment if the bacterial species is known. Classification accuracies can thus be condensed into a new confusion matrix grouped by empiric antibiotic treatment (Fig. 2b), where the average accuracy of our method is 97.0±0.3%. In comparison, logistic regression and SVM achieve accuracies of 93.3% and 92.2%, respectively.

**Fig. 3:**
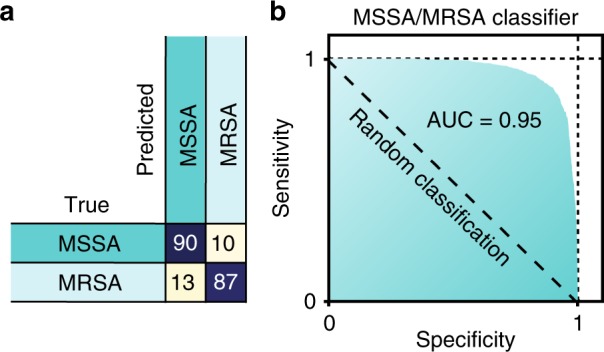
Binary MRSA/MSSA classifier. **a** A binary classifier is used to distinguish between methicillin-resistant and -susceptible *S. aureus* (MRSA/MSSA), achieving 89.1±0.1% accuracy. **b** By varying the classification threshold, it is possible to trade off between sensitivity (true positive rate) and specificity (true negative rate). The ROC curve shows sensitivities and specificities significantly higher than random classification, with an AUC of 0.953

Beyond empiric first choice antibiotics, clinicians also conduct antibiotic susceptibility tests to determine bacterial responses to drugs. As a step toward a culture-free antibiotic susceptibility test using Raman spectroscopy, we train a binary CNN classifier to differentiate between methicillin-resistant and -susceptible isolates of *S. aureus*. This model achieves 89.1±0.1% identification accuracy (Fig. 3a). Because the consequences for misdiagnosing MRSA as MSSA are often more severe than the reverse misdiagnosis, the binary decision can be tuned for higher sensitivity (low false negative rate), as shown in the receiver operating characteristic (ROC) curve in Fig. 3b (dotted line denotes performance of random guessing). The area under the curve (AUC) is 0.953, meaning that a randomly selected positive example (i.e., Raman sample from patient with MRSA) will be predicted to be more likely to be MRSA than a randomly selected negative example (i.e., sample from patient with MSSA) with probability 0.953.

### Extension to clinical patient isolates

**Fig. 4:**
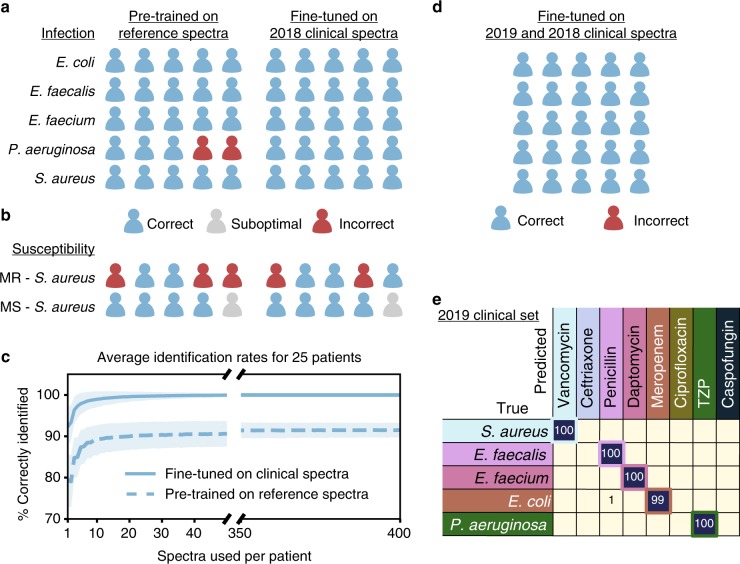
Extension to clinical patient isolates. A CNN pre-trained on our reference dataset can be extended to classify clinical patient isolates and further improved by fine-tuning on a small number of clinical spectra. **a** 5 species of bacterial infections are tested, with 5 patients per infection type. Each patient is classified into one of 8 treatment classes where each species corresponds to a different treatment class. After fine-tuning, species identification accuracy improves from 89.0±3.6% to 99.0±1.9% (± calculated as standard deviation across 10,000 sampling trials). **b** Binary classification between MRSA and MSSA patient isolates is also performed, with an accuracy of 61.7±7.3% that improves to 65.4±6.3% after fine-tuning. **c** Dependence of average diagnosis rates for the fine-tuned model on the number of spectra used per patient. With just 10 spectra, the performance of the model reaches 99% — within 1% difference of the performance with 400 spectra (100%). Error bars are calculated as the standard deviation across 10,000 trials of random selections of n spectra, where n is the number of spectra used per patient. **d** We perform an additional test on a new clinical dataset gathered from an additional 25 patients with the same distribution across species as the first clinical dataset. We update the model that is pre-trained on the reference dataset and fine-tuned on the first clinical dataset by fine-tuning on the second clinical dataset using the same procedure. **e** Detailed breakdown by class for the second clinical dataset. Correct pairings between species and treatment group are outlined in the colored boxes. The rate of accurate identification is 99.7±1.1%

To demonstrate that this approach can be extended to new clinical settings, we test our model on two groups of 25 clinical isolates derived from patient samples, for a total of 50 patients, Within each patient group, samples include 5 isolates from each of the 5 most prevalent^39^ empiric treatment groups (see Supplementary Table 2 and Supplementary Fig. 4). We first consider isolates from 25 patients collected from Palo Alto VA Medical Center in 2018. We augment our reference dataset with this clinical dataset comprised of 400 spectra per clinical isolate. To account for changes in the relative prevalence of species and antibiotic resistances over time, the model may be fine-tuned on a small dataset that is representative of current patient populations. We use a leave-one-patient-out cross-validation (LOOCV) strategy for fine-tuning, where we assign 1 patient in each class to the test set (5 patients total) and use the other 4 for fine-tuning (20 patients total), fine-tuning on 10 randomly sampled spectra per patient isolate — we repeat this process 5 times, so all 25 patient isolates appear in the held-out test set once. We then use 10 randomly sampled spectra from each patient isolate in the test set to reach an infection identification for that patient isolate. The sampling procedure for identification is repeated for 10,000 trials, and we report the average accuracy and standard deviation, and display a trial representing the modal result in Fig. 4a (full experiment details can be seen in Supplementary Note 1). A CNN pre-trained on the reference dataset serves both as initialization for the fine-tuned model and as a baseline, achieving 89.0±3.6% (± calculated as standard deviation across 10,000 sampling trials) species identification accuracy, a statistically significant improvement over logistic regression and support vector machine baselines (see Methods for details). When the CNN is fine-tuned on clinical data and then evaluated on the held-out patients, the identification accuracy is improved to 99.0±1.9% (Supplementary Fig. 5). Samples for the clinical tests were prepared separately for each patient, so we conclude that the measured performance is not due to batch effects from sample preparation or measurement conditions.

Because patient samples may contain very low numbers of bacterial cells without culturing (e.g. 1 CFU/mL or fewer in blood^40^), only a few individual bacterial spectra per patient may be available to make a diagnosis. As seen in Fig. 4c, just 10 cellular spectra are enough to reach high identification accuracy. The rate of correct identification using 10 spectra is 99.0%, within 1% of the performance with 400 spectra (100.0%). While acquiring spectra from 400 individual bacterial cells would likely necessitate culturing, we achieve high accuracy on spectra from 10 individual bacterial cells, commensurate with typical levels of bacterial cells present in uncultured samples^40,41^.

For a proof-of-concept antibiotic susceptibility test on clinical isolates, we collect Raman spectra on 5 additional clinical MRSA isolates and test the binary MRSA/MSSA classifier that is pre-trained on the reference MRSA and MSSA isolates. Using the same LOOCV process, we fine-tune the binary classifier on the clinical spectra. A representative result is shown in Fig. 4b; any misclassifications of MSSA as MRSA are labeled as “suboptimal”, indicating that Vancomycin (prescribed for MRSA) is also effective on MSSA but is not considered optimal treatment and may introduce adverse patient effects. On average, the pre-trained binary classifier achieves 61.7$\pm$7.3% accuracy and the fine-tuned binary classifier achieves 65.4$\pm$6.3% accuracy (Supplementary Fig. 5).

Finally, to test the robustness of the fine-tuning approach over multiple clinical datasets, we use our second patient group of 25 isolates, collected from Stanford Hospital from February 2019 to March 2019. We conduct additional fine-tuning of the model that is pre-trained on the reference dataset and fine-tuned on the original clinical dataset. The treatment group identification accuracy on the new clinical dataset using only 10 spectra per patient is 99.7±1.1% Fig. 4d, e, with improved performance for both *S. aureus* and *P. aeruginosa*, demonstrating the potential for continuous improvement of the trained model.

## Discussion

In this work, we apply state-of-the-art deep learning techniques to noisy Raman spectra to identify clinically relevant bacteria and their empiric treatment. A CNN model pre-trained on our dataset can easily be extended to new clinical settings through fine-tuning on a small number of clinical isolates, as we have shown on our clinical dataset. We envision that fine-tuning processes such as the one demonstrated here could be important components for continuously evaluating and improving deployed models. Our model, applied here to the identification of clinically relevant bacteria, can be applied with minimal modification to other identification problems such as materials identification, or other spectroscopic techniques such as nuclear magnetic resonance, infrared, or mass spectrometry.

This study uses measurement times of 1 s, corresponding to SNRs that are an order of magnitude lower than typical reported bacterial spectra — while still achieving comparable or improved identification accuracy on more isolate classes than typical Raman bacterial identification studies. A common strategy for reducing measurement times is surface-enhanced Raman scattering (SERS) using plasmonic structures, which can increase the signal strength by several orders of magnitude^11,42,43^. SERS spectra can be highly variable and difficult to reproduce, particularly on cell samples^8,44^, making it difficult to develop a reliable diagnostic method based on SERS. However, with a dataset capturing the breadth of variation in SERS spectra, a CNN could enable a platform that processes blood, sputum, or urine samples in a few hours.

Compared to other culture-free methods^45^ including single-cell sequencing^46–49^ and fluorescence or magnetic tagging^50^, Raman spectroscopy has the unique potential to be a technique for identifying phenotypes that does not require specially designed labels, allowing for easy generalizability to new strains.

To achieve treatment recommendations as fine-grained as those from culture-based methods, larger datasets covering more resistant and susceptible clinical isolates, greater diversity in antibiotic susceptibility profiles, cell states, and growth media and conditions would be needed. Though collecting such datasets is beyond an academic scope, requiring highly automated sample preparation and data acquisition processes, there is promise for clinical translation. Similarly, studies applying the Raman-CNN system to identify pathogens in relevant biofluids such as whole blood, sputum, and urine are a promising future direction to demonstrate the validity of the method as a diagnostic tool. When combined with such an automated system, the Raman-CNN platform presented here could rapidly scan and identify every cell in a patient sample and recommended an antibiotic treatment in one step, without needing to wait for a culture step. Such a technique would allow for accurate and targeted treatment of bacterial infections within hours, reducing healthcare costs and antibiotics misuse, limiting antimicrobial resistance, and improving patient outcomes.

## Methods

### Dataset

The reference dataset consists of 30 bacterial and yeast isolates, including multiple isolates of Gram-negative and Gram-positive bacteria, as well as Candida species. We also include an isogenic pair of *S. aureus* from the same strain, in which one variant contains the *mecA* resistance gene for methicillin (MRSA) and the other does not (MSSA)^51^ (see Supplementary Table 1 for full isolate information). The reference training dataset consists of 2000 spectra each for the 30 reference isolates plus isogenic MSSA at 3 measurement times. The reference fine-tuning and test datasets each consist of 100 spectra for each of the 30 reference isolates. The first clinical dataset consists of 30 patient isolates distributed across 5 species, with 400 spectra per isolate. The second clinical dataset consists of 25 patient isolates distributed across the same 5 species, with 100 spectra per isolate. Due to degradation in optical system efficiency, the measurement times for the reference fine-tuning and test and second clinical datasets were increased from 1 s to 2 s in order to keep SNR consistent across datasets. Antibiotic susceptibility was performed by first genotypic testing for methicillin by detecting mecA using PCR (PMID: 19741081). Then phenotypic antimicrobial susceptibility testing was performed on the Microscan Walkaway instrument (Beckman Coulter, Brea, CA) and VITEK® 2 (Biomérieux, Inc., Durham, NC).

### Dataset variance

For our datasets, we observe that intra-sample variance is high, as demonstrated by the pairwise spectral difference analysis summarized in Supplementary Fig. 2. For 19 out of 30 isolates, spectra from at least one other isolate are more similar on average than spectra from the same isolate, on average. For example, when we rank isolates in order of similarity to *E. faecalis* 2 (Supplementary Fig. 2c), there are 8 other isolates where the average difference between a spectrum from *E. faecalis* 2 and a spectrum from the other isolate is smaller than the average difference between two spectra from *E. faecalis* 2. When intra-sample variance is high, a large number of spectra per sample may help to better represent the full data distribution and lead to higher predictive performance.

### Sample preparation

Bacterial isolates were cultured on blood agar plates each day before measurement. Plates were sealed with Parafilm and stored at 4 °C for 20 min to 12 h before sample preparation. Storage times varied to allow for multiple measurement times per day; however all other sample preparation conditions were kept consistent between samples. Differences in storage time were not found to result in spectral changes greater than spectral changes due to strain or isogenic differences. All clinical isolates were prepared in separate samples with consistent sample preparation conditions. Because test samples were prepared separately from samples used for training, we conclude that classifications are not due to batch effects such as differences in sample preparation. We prepared samples for measurement by suspending 0.6 mg of biomass from a single colony in 10  µL of sterile water (0.4 mg in 5  µL water for Gram-positive species) and drying 3 µL of the suspension on a gold-coated silica substrate (Fig. 1a, b). Substrates were prepared by electron beam evaporation of 200 nm of gold onto microscope slides that were pre-cleaned using base piranha. Samples were allowed to dry for 1 h before measurement.

### Raman measurements

We measured Raman spectra across monolayer regions of the dried samples (Fig. 1a) using the mapping mode of a Horiba LabRAM HR Evolution Raman microscope. 633 nm illumination at 13.17 mW was used with a 300 l/mm grating to generate spectra with 1.2 cm^−1^ dispersion to maximize signal strength while minimizing background signal from autofluorescence. Wavenumber calibration was performed using a silicon sample. The ×100 0.9 NA objective lens (Olympus MPLAN) generates a diffraction-limited spot size, $\sim$1  µm in diameter. A 45 × 45 discrete spot map is taken with 3  µm spacing between spots to avoid overlap between spectra. The spectra are individually background corrected using a polynomial fit of order 5 using the subbackmod Matlab function available in the Biodata toolbox (see Supplementary Fig. 1 for examples of raw and corrected spectra). The majority of spectra are measured on true monolayers and arise from ~1 cell due to the diffraction-limited laser spot size, which is roughly the size of a bacteria cell. However, a small number of spectra may be taken over aggregates or multilayer regions. We exclude the spectra that are most likely to be non-monolayer measurements by ranking the spectra by signal intensity and discarding the 25 spectra with highest intensity, which includes all spectra with intensities greater than two standard deviations from the mean. We measured both monolayers and single cells, and found that monolayer measurements have SNRs of 2.5 ± 0.7, similar to single-cell measurements (2.4 ± 0.6), while allowing for the semi-automated generation of a large training dataset. The spectral range between 381.98 and 1792.4 cm^−1^ was used, and spectra were individually normalized to run from a minimum intensity of 0 and maximum intensity of 1 within this spectral range. SNR values are calculated by dividing the total intensity range by the intensity range over a 20-pixel wide window in a region where there is no Raman signal.

### CNN architecture & training details

The CNN architecture is adapted from the Resnet architecture^37^ that has been widely successful across a range of computer vision tasks. It consists of an initial convolution layer followed by 6 residual layers and a final fully connected classification layer — a block diagram can be seen in Fig. 1. The residual layers contain shortcut connections between the input and output of each residual block, allowing for better gradient propagation and stable training (refer to reference 37 for details). Each residual layer contains 4 convolutional layers, so the total depth of the network is 26 layers. The initial convolution layer has 64 convolutional filters, while each of the hidden layers has 100 filters. These architecture hyperparameters were selected via grid search using one training and validation split on the isolate classification task. We also experimented with simple MLP (multi-layer perceptron) and CNN architectures but found that the Resnet-based architecture performed best.

We first train the network on the 30-isolate classification task, where the output of the CNN is a vector of probabilities across the 30 classes and the maximum probability is taken as the predicted class. The binary MRSA/MSSA and binary isogenic MRSA/MSSA classifiers have the same architecture as the 30-isolate classifier, aside from the number of classes in the final classification layer. We use the Adam optimizer^52^ across all experiments with learning rate 0.001, betas (0.5, 0.999), and batch size 10. Classification accuracies are reported across 5 randomly selected train and validation splits. We first pre-train the CNN on the reference training dataset, then fine-tune on the reference fine-tuning dataset to account for measurement changes due to degradation in optical system efficiency. For each of the 5 splits, we split the fine-tuning data into 90/10 train and validation splits, train the CNN on the train split, and use the accuracy on the validation split to perform model selection. We then evaluate and report the test accuracy on the test dataset which is gathered from independently cultured and prepared samples. The binary MRSA/MSSA classifier is trained and fine-tuned using the same procedure. The binary isogenic MRSA/MSSA classifier is trained using a similar procedure on data from a single measurement series.

All error values reported for tests on the reference dataset are standard deviation values across 5 splits.

While a high number of samples is good for ensuring dataset variation, deep learning approaches can still benefit from having a high number of examples per sample. When intra-sample variance is high, as we observe for our datasets, a large number of spectra per sample may better represent the full distribution and lead to higher predictive performance.

For the clinical isolates, we start by pre-training a CNN on the empiric treatment labels for the 30 reference isolates. We then use the following leave-one-patient-out cross-validation (LOOCV) strategy to fine-tune the parameters of the CNN. There are a total of 25 patient isolates across 5 species. In each of the 5 folds, we assign 1 patient in each species to the test set, 1 patient in each species to the validation set, and the remaining 3 patients in each species to the training (i.e., fine-tuning) set. We then use the clinical training set (consisting of isolates from 15 patients) to fine-tune the CNN parameters, and use accuracy on the validation set (5 patient isolates) to do model selection. The test accuracy for each fold is evaluated on the test set (5 patient isolates) using the method described below.

### Clinical identification data analysis

To reach an identification for patient isolates, 400 spectra are measured across a sample from each patient isolate. 10 of these spectra are chosen at random to be classified. The most common class out of the 10 spectral classifications is then chosen as the identification for each patient isolate, with ties broken randomly. All error values reported for tests on the clinical dataset are standard deviations across 10,000 trials of random selections of 10 spectra, with an upper accuracy bound of 100%. For the second clinical dataset, we perform the same procedure, except that we choose 10 out of 100 spectra for each patient isolate, and use a model that is both pre-trained on the reference dataset and fine-tuned on the first clinical dataset.

### Baselines

In all experiments where logistic regression (LR) and support vector machine (SVM) baselines were used, we first used PCA to reduce the input dimension from 1000 to 20 — this hyperparameter was determined by plotting test accuracies for different settings on one training and validation split for the 30 isolate task and picking a value near where the test accuracy saturated. Using only the first 20 principal components not only decreases computation costs, but also increases accuracy by reducing the amount of noise in the data. For each fold of the cross validation procedure, we use grid search to choose the regularization hyperparameter for each model achieving the best validation accuracy and report the corresponding test accuracy. Using both the training and fine-tuning reference datasets to train the baseline models, LR and SVM achieve 57.5% and 56.8% on the 30-class task and 89.0% and 88.3% on the empiric treatment task, respectively. Using only the fine-tuning reference dataset, LR and SVM achieve 75.7% and 74.9% on the 30-class task and 93.3% and 92.2% on the empiric treatment task, respectively. The latter performance is higher because the baseline models do not benefit from additional training data as the CNN does, but rather benefit from training data the most closely matches the measurement conditions of the test data.

### Two-sample test of sample means

We use the Welch’s two-sample $t$-test to test whether the differences in mean clinical accuracy for the CNN and the SVM and LR baselines were statistically significant. Welch’s $t$-test is a variation of the Student’s $t$-test that is used when the two samples may have unequal variances. In each case, we start by computing the pooled standard deviation as

1 $$\sigma =\sqrt{\frac{({n}_{1}-1){\sigma }_{1}^{2}+({n}_{2}-1){\sigma }_{2}^{2}}{{n}_{1}+{n}_{2}-2}}.$$

We then compute the standard error of the difference between the means as

2 $${\rm{se}}=\sigma \times \sqrt{\frac{1}{{n}_{1}}+\frac{1}{{n}_{2}}}.$$

Finally, we can compute the test statistic as

3 $$t=\frac{{\mu }_{1}-{\mu }_{2}}{{\rm{se}}},$$

and then compute the p-value using the corresponding Student’s $t$-distribution. For our computations, ${n}_{{\rm{CNN}}}={n}_{{\rm{LR}}}={n}_{{\rm{SVM}}}=10000$, ${\mu }_{{\rm{CNN}}}=89.0$, ${\mu }_{{\rm{LR}}}=81.8$, ${\mu }_{{\rm{SV M}}}=82.9$, ${\sigma }_{{\rm{CNN}}}=3.6$, ${\sigma }_{{\rm{LR}}}=6.0$, and ${\sigma }_{{\rm{SV M}}}=5.9$. In comparing the CNN with LR, we computed a $t$-statistic of 102.9 and in comparing the CNN with SVM, we computed a $t$-statistic of 88.3. In both cases, we reject the null hypothesis that the means are equal at the 1e-6 p-level.

### Biological materials availability

Unique isolates are available from the authors upon reasonable request.

### Reporting Summary

Further information on research design is available in the Nature Research Reporting Summary linked to this article.

## Supplementary Information

Supplementary Information

Reporting Summary

## Data Availability

All data needed to replicate these results are available at https://github.com/csho33/bacteria-ID.

## Code availability

All code needed to replicate these results is available at https://github.com/csho33/bacteria-ID.
