# Supplementary material for: Rapid identification of pathogenic bacteria using Raman spectroscopy and deep learning
Source: Nat Commun. 2019 Oct 30;10:4927. doi: 10.1038/s41467-019-12898-9 (PMC6960993; doi:10.1038/s41467-019-12898-9)
Supplement: Supplementary file 1 — Supplementary Information [file 41467_2019_12898_MOESM1_ESM.pdf]

Supplementary Information:

Rapid identification of pathogenic bacteria using Raman spectroscopy and deep learning

Ho *et al*.

**Species**	**Figure label**	**Isolate code**	**Empiric antibiotic treatment**
Escherichia coli	*E. coli* 1	ATCC 25922	Meropenem
Escherichia coli	*E. coli* 2	ATCC 700728	Meropenem
Klebsiella pneumoniae	*K. pneumoniae* 1	ATCC 33495	Meropenem
Klebsiella pneumoniae	*K. pneumoniae* 2	Stanford Clinical Collection	Meropenem
Klebsiella aerogenes	*K. aerogenes*	ATCC 13048	Meropenem
Enterobacter cloacae	*E. cloacae*	ATCC 13047	Meropenem
Proteus mirabilis	*P. mirabilis*	ATCC 43071	Meropenem
Serratia marcescens	*S. marcescens*	ATCC 13880	Meropenem
Pseudomonas aeruginosa	*P. aeruginosa* 1	ATCC 27853	Meropenem
Pseudomonas aeruginosa	*P. aeruginosa* 2	ATCC 9027	Meropenem
Staphylococcus aureus	MSSA 1	ATCC 25923	Vancomycin
Staphylococcus aureus	MSSA 2	ATCC 6538	Vancomycin
Staphylococcus aureus	MSSA 3	ATCC 29213	Vancomycin
Staphylococcus epidermidis	*S. epidermidis*	ATCC 12228	Vancomycin
Staphylococcus lugdunensis	*S. lugdunensis*	ATCC 49576	Vancomycin
Staphylococcus aureus	isogenic MSSA	USA300-ex	Vancomycin
Staphylococcus aureus	MRSA 1 (isogenic)	USA300-wt	Vancomycin
Staphylococcus aureus	MRSA 2	ATCC 43300	Vancomycin
Streptococcus pneumoniae	*S. pneumoniae* 1	ATCC 49619	Ceftriaxone
Streptococcus pneumoniae	*S. pneumoniae* 2	ATCC 6305	Ceftriaxone
Streptococcus pyogenes (Group A)	Group A *Strep.*	ATCC 19615	Penicillin
Streptococcus agalactiae (Group B)	Group B *Strep.*	ATCC 12386	Penicillin
Streptococcus dysgalactiae (Group C)	Group C *Strep.*	ATCC 12388	Penicillin
Streptococcus dysgalactiae (Group G)	Group G *Strep.*	ATCC 12394	Penicillin
Streptococcus sanguinis	*S. sanguinis*	ATCC 35571	Penicillin
Enterococcus faecalis	*E. faecalis* 1	ATCC 29212	Penicillin
Enterococcus faecalis	*E. faecalis* 2	ATCC 51299	Penicillin
Enterococcus faecium	*E. faecium*	ATCC 700221	Daptomycin
Salmonella enterica	*S. enterica*	ATCC 13314	Ciprofloxacin
Candida albicans	*C. albicans*	ATCC 10231	Caspofungin
Candida glabrata	*C. glabrata*	ATCC 66032	Caspofungin

Supplementary Table 1: Reference isolates. The empiric treatments are chosen by the authors of this paper specializing in infectious diseases from recommendations from Sanford Guide to Antimicrobial Therapy and trends in patient susceptibility profiles at the Stanford Hospital and the Veterans Affairs Palo Alto Health Care System [1,2]. However, specific choices for each of the empiric species groups may be modified according to individual hospital susceptibility profiles.

Species	Figure label	Source	Isolate code
Escherichia coli	*E. coli*	Urine Culture	1136
Escherichia coli	*E. coli*	Sputum	1881
Escherichia coli	*E. coli*	Urine Culture	1923
Escherichia coli	*E. coli*	Urine Culture	1925
Escherichia coli	*E. coli*	Tissue	1959
Pseudomonas aeruginosa	*P. aeruginosa*	Peritoneal Fluid	1964
Pseudomonas aeruginosa	*P. aeruginosa*	Sputum	2012
Pseudomonas aeruginosa	*P. aeruginosa*	Sputum	2043
Pseudomonas aeruginosa	*P. aeruginosa*	Sputum	2044
Pseudomonas aeruginosa	*P. aeruginosa*	Wound	2046
Staphylococcus aureus	*S. aureus*/MSSA	Blood Culture	0608
Staphylococcus aureus	*S. aureus*/MSSA	Blood Culture	2142
Staphylococcus aureus	*S. aureus*/MSSA	Blood Culture	5293
Staphylococcus aureus	*S. aureus*/MSSA	Blood Culture	6007
Staphylococcus aureus	*S. aureus*/MSSA	Blood Culture	8987
Staphylococcus aureus	MRSA	Sputum	0341
Staphylococcus aureus	MRSA	Blood Culture	0342
Staphylococcus aureus	MRSA	Urine Culture	0343
Staphylococcus aureus	MRSA	Wound	0344
Staphylococcus aureus	MRSA	Sputum	0389
Enterococcus faecalis	*E. faecalis*	Pleural Fluid	1613
Enterococcus faecalis	*E. faecalis*	Blood Culture	1698
Enterococcus faecalis	*E. faecalis*	Abscess, Abdominal	1797
Enterococcus faecalis	*E. faecalis*	Tissue	1899
Enterococcus faecalis	*E. faecalis*	Blood Culture	1903
Enterococcus faecium	*E. faecium*	Urine Culture	1980
Enterococcus faecium	*E. faecium*	Urine Culture	1981
Enterococcus faecium	*E. faecium*	Tissue	1985
Enterococcus faecium	*E. faecium*	Urine Culture	1986
Enterococcus faecium	*E. faecium*	Urine Culture	1992

Supplementary Table 2: Clinical isolates

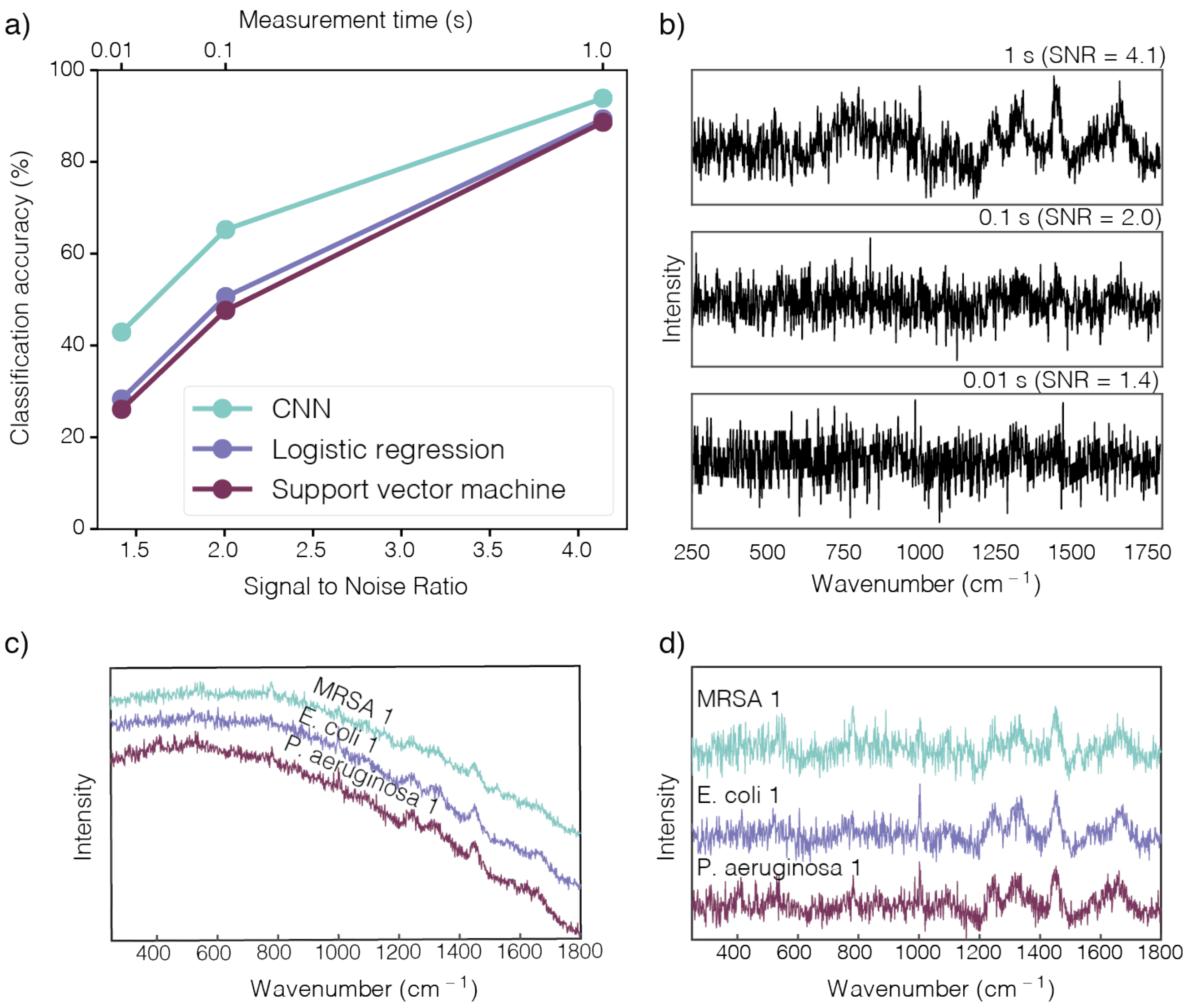

Supplementary Figure 1: a) Isolate-level classification accuracy increases with SNR. Under the measurement conditions used in this study, performance of the CNN is negatively affected by shorter measurement times. Further increase of SNR should saturate the performance of the CNN to a minimal baseline error rate. For this experiment, training, validation, and test sets are split between a single measurement series for each isolate. b) Spectral examples (from *E. coli* 1) for measurement times of 1 s, 0.1 s, and 0.01 s. c) Raw spectra for MRSA 1, *E. coli* 1, and *P. aeruginosa* 1 for a measurement time of 1 s. d) Spectra after background subtraction and normalization for a measurement time of 1 s. These are the direct inputs into our model.

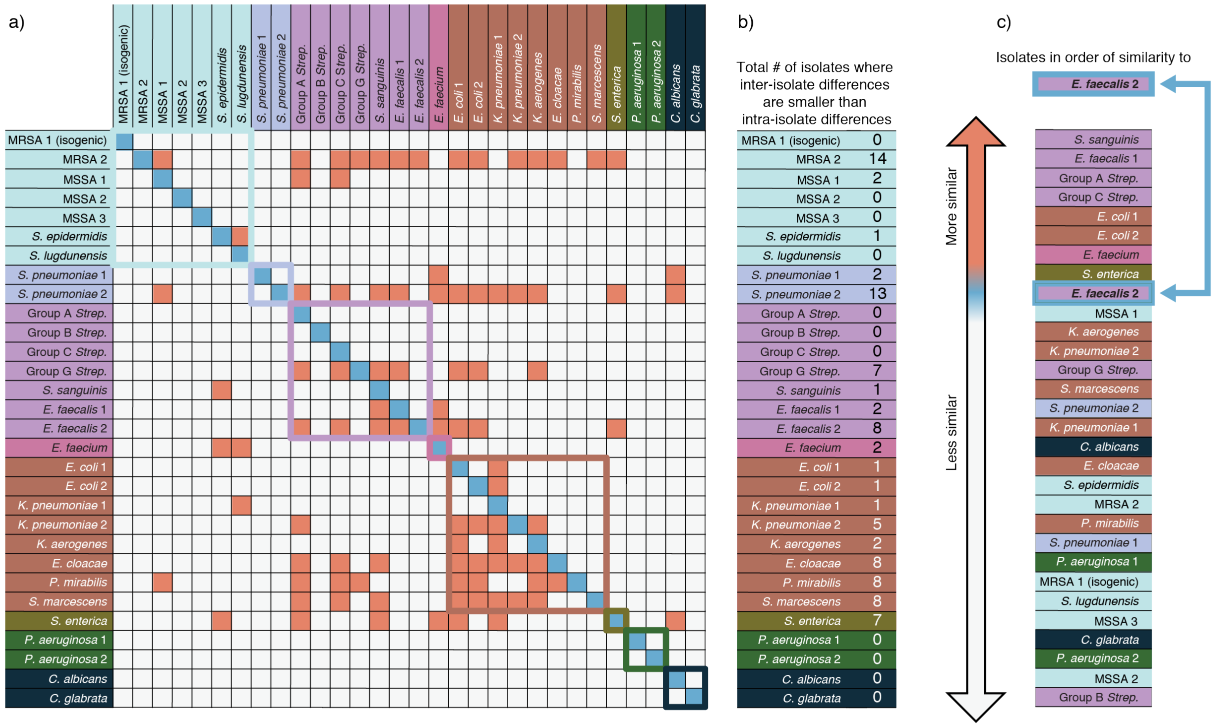

Supplementary Figure 2: Inter-isolate vs intra-isolate pairwise spectral differences. Average differences are calculated as the average L2 distance between pairs of spectra over 4 million (2000 x 2000) possible pairs. a) Intra-isolate distances (along the diagonal) are computed as the difference between two spectra from the same isolate, while inter-isolate distances (off-diagonals) are computed as the difference between one spectrum from the row isolate and one spectrum from the column isolate. For each row, red marks indicate isolates for which inter-isolate differences are smaller than the average intra-isolate difference for the isolate in that row. Blue marks simply indicate the location of the diagonal for reference. For example, in the second row, the average distance between an MSSA 1 spectrum and an MRSA 1 spectrum is smaller than the average distance between two MRSA 1 spectra in other words, MSSA 1 and MRSA 1 spectra are more similar (on average) than MRSA 1 spectra are to themselves (on average). b) For each isolate, we summarize the total number of more similar isolates. For 19 out of 30 isolates, spectra from at least one other isolate are more similar than spectra from the same isolate. c) Example sort by similarity for E. faecalis 2, demonstrating that spectra from 8 isolates are more similar on average to E. faecalis 2 than different spectra from E. faecalis itself, on average.

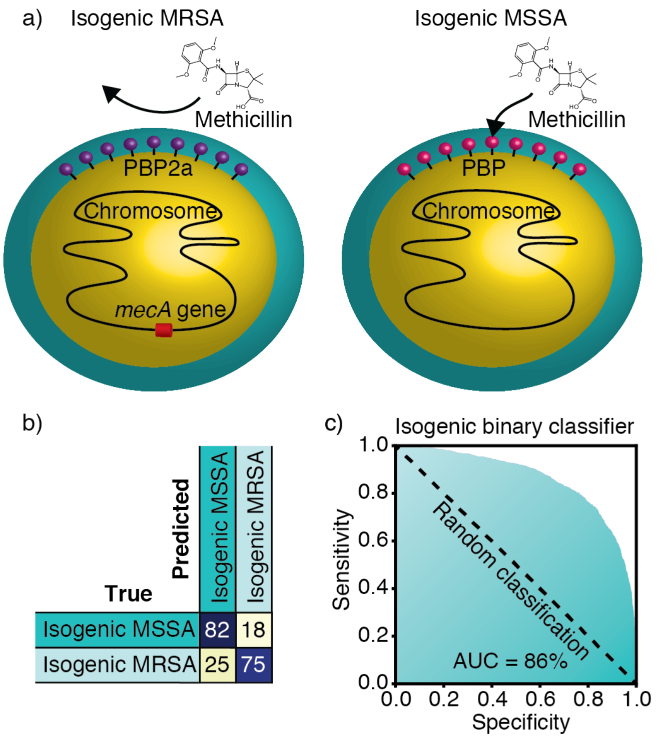

Supplementary Figure 3: Isogenic MRSA/MSSA classifier. a) Sensitivity to antibiotic resistance alone with all other factors held constant can be tested using an isogenic pair of *S. aureus*, meaning that the two are genetically identical aside from the deletion of the *mecA* gene which confers methicillin resistance[3]. The expression of *mecA* results in replacement of Penicillin Binding Proteins (PBPs) with PBP2a, which has a low binding affinity for methicillin. b) A binary classifier is trained to distinguish between MRSA 1 and its isogenic variant, achieving 78.5±0.6% accuracy. For this experiment, training, validation, and test sets are split between a single measurement series for each isolate. These results are a first step in ongoing work aiming to understand whether isogenic pairs can be distinguished by their Raman spectra. Because the measured spectral differences are so small between isogenic pairs, we expect that true signal differences may be confounded by experimental factors including minute differences in sample drying time, incubation time, and sample positioning. These confounding factors would need to be carefully controlled for in future experiments where training, validation, and test sets are split between independently cultured and prepared samples. c) The ROC shows sensitivities and specificities significantly higher than random classification, with an AUC of 86.1%.

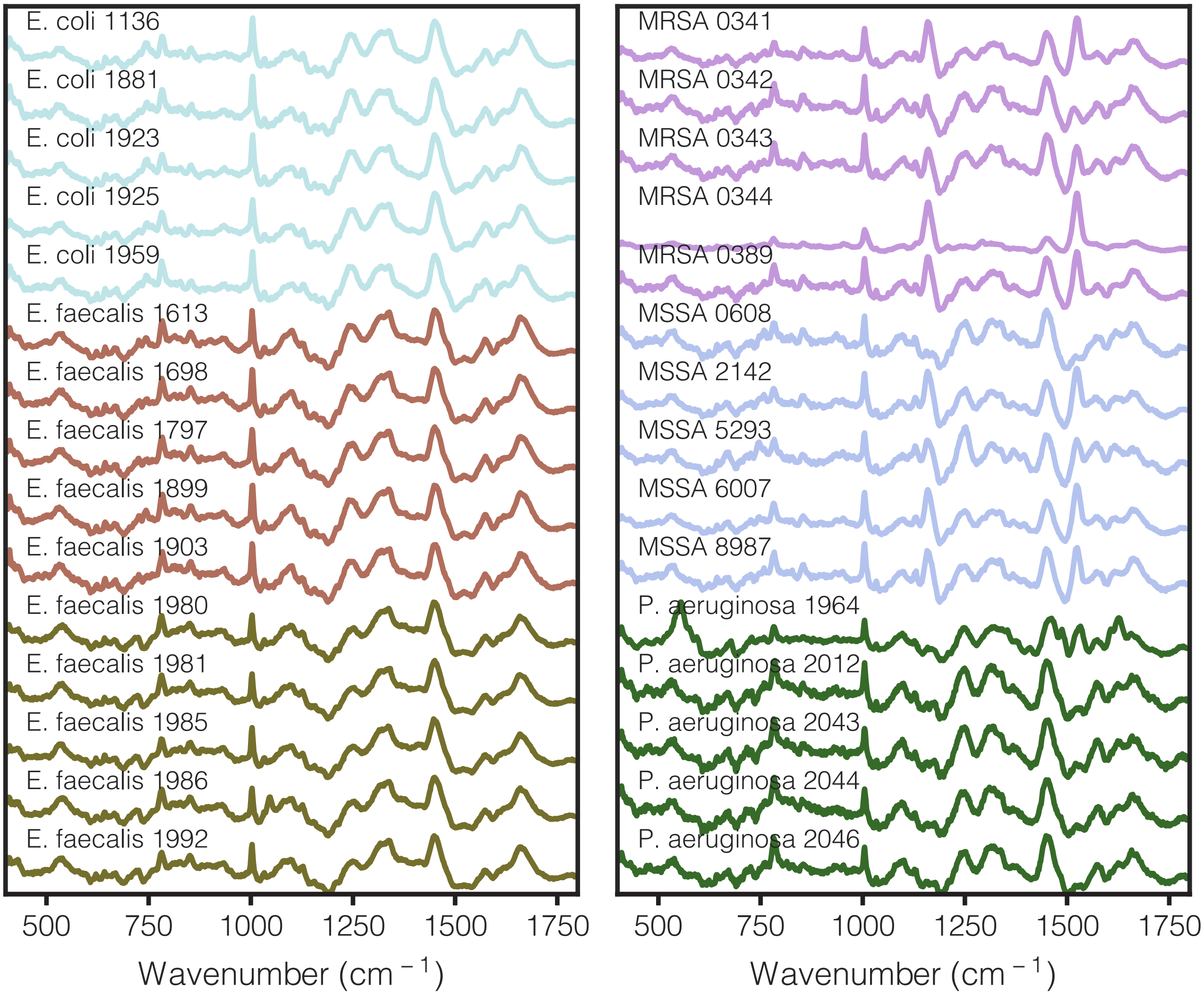

Supplementary Figure 4: Spectra for individual patient isolates, averaged across the full 400 spectra dataset for each patient.

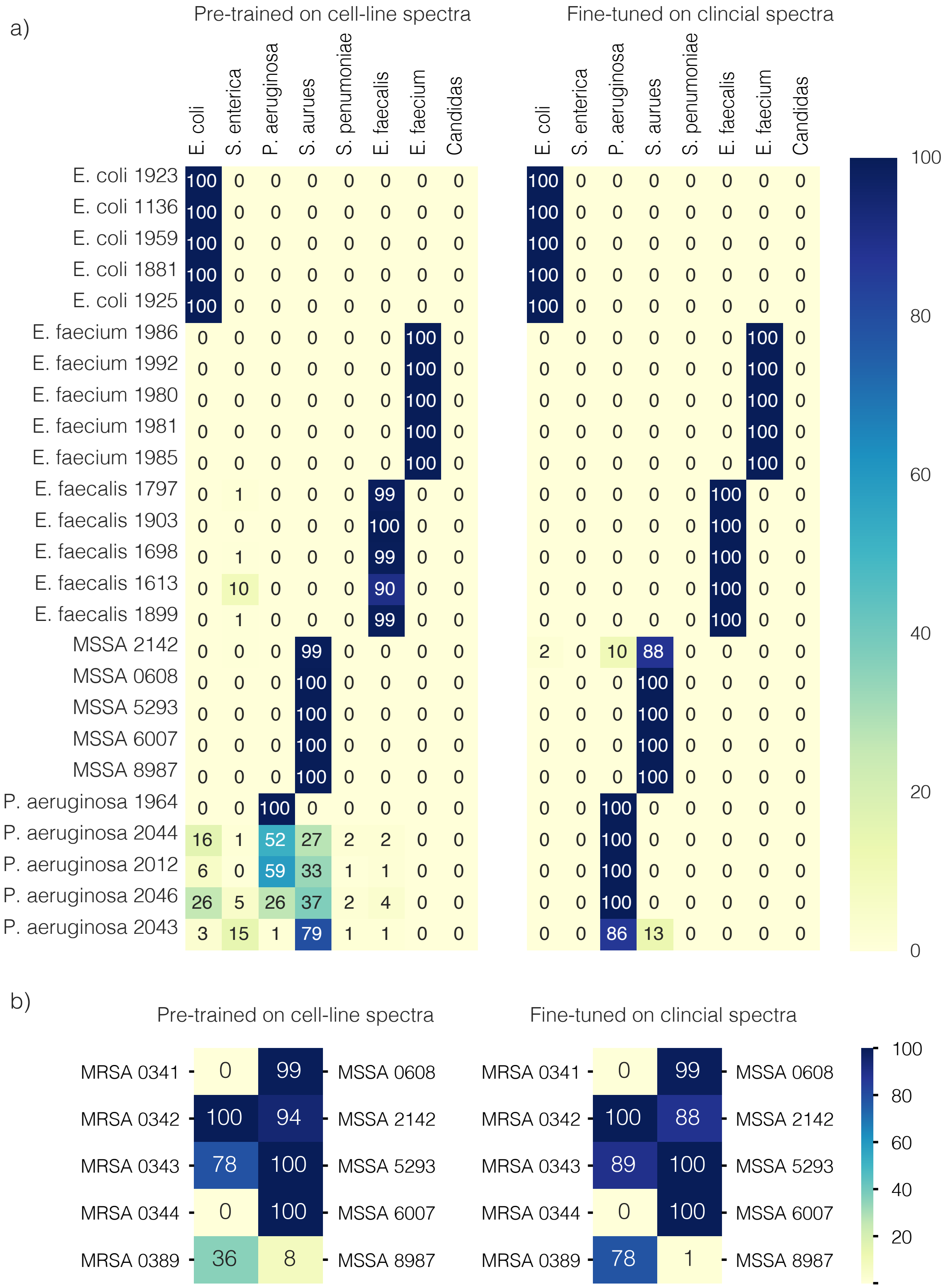

Supplementary Figure 5: a) Classification results for each patient isolate. Element (i, j) represents the percentage out of 10,000 trials in which species j is predicted by the CNN for patient i. b)Classification results for each MRSA/MSSA patient isolate. Heatmap represents the percentage out of 10,000 trials in which the binary CNN accurately identifies whether the isolate is MRSA or MSSA. 10 spectra per isolate are used for both fine tuning and identification.

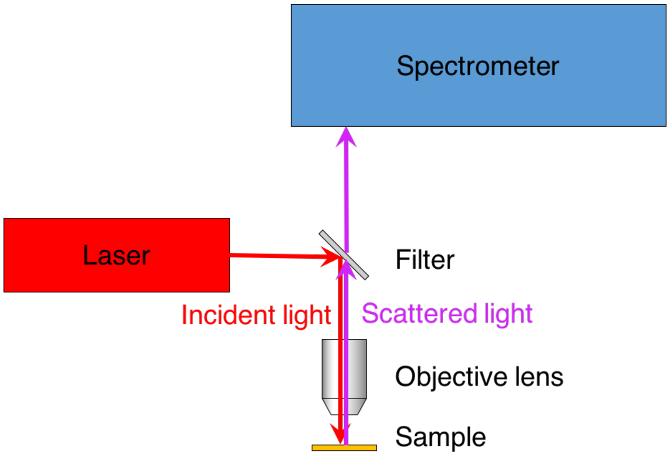

Supplementary Figure 6: Experimental schematic of the Horiba Labram Raman spectrometer.

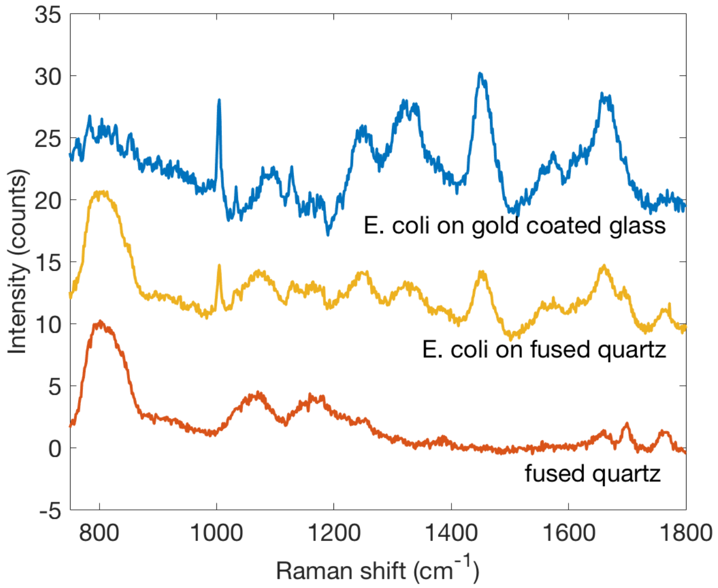

Supplementary Figure 7: Comparison of signal intensity on reflective and non-reflective substrates. We find that the signal intensity and SNR of our measurements on gold substrates is 2X the the signal intensity and SNR of measurements on glass substrates. Because quartz is transparent at visible wavelengths and gold is reflective, it is more likely that this 2X enhancement is due to the reflection of forward-scattered photons rather than a SERS enhancement. These measurements were taken with the same measurement conditions as our datasets, but consist of 100 1s accumulations to help visualize the spectral shape with less noise.

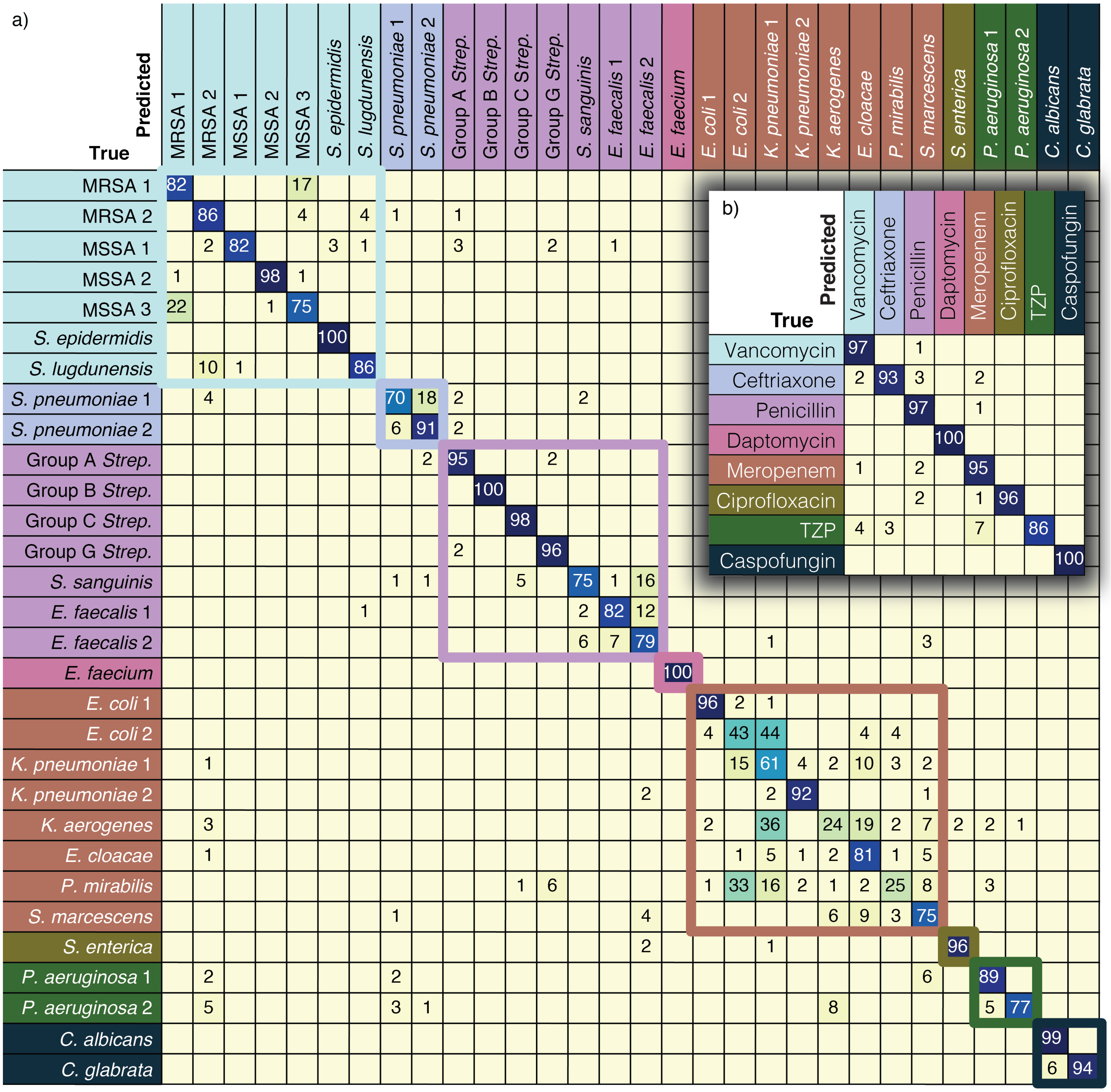

Supplementary Figure 8: CNN performance breakdown by class with test and fine-tune datasets swapped. The trained CNN classifies 30 bacterial and yeast isolates with isolate-level accuracy of 81.6±0.6% and antibiotic grouping-level accuracy of 95.9±0.6%. a) Confusion matrix for 30 strain classes. Entry (i, j) represents the percentage out of 100 test spectra that are predicted by the CNN as class j given a ground truth of class i; entries along the diagonal represent the accuracies for each class. Misclassifications are mostly within antibiotic groupings, indicated by colored boxes, and thus do not affect the treatment outcome. b) Predictions can be combined into antibiotic groupings to estimate treatment accuracy. TZP = piperacillin-tazobactam. All values below 0.5% are not shown.

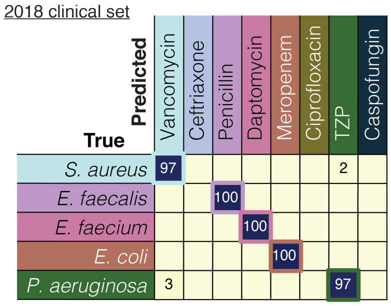

Supplementary Figure 9: Detailed breakdown by class for the first clinical dataset. Each patient is classified into one of 8 treatment classes where each species corresponds to a different treatment class. Correct pairings between species and treatment group are outlined in the colored boxes. The rate of accurate identification is 99.0±1.9%.

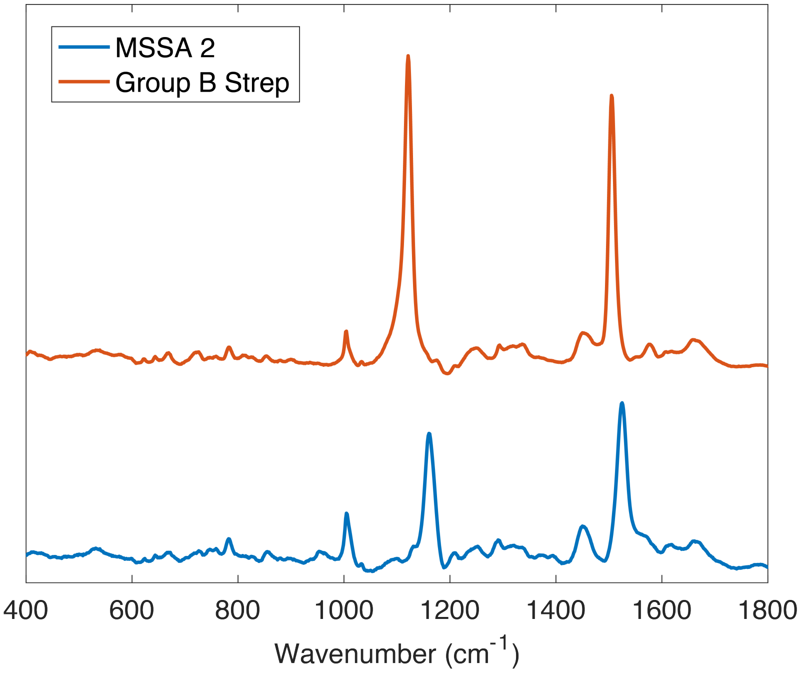

Supplementary Figure 10: The spectra of MSSA 2 and Group B *Strep.* demonstrate resonant Raman effects from chromophores (e.g. carotenoids or cytochromes), resulting in enhanced Raman peaks around 1005 cm^{-1}, 1121-1162 cm^{-1}, and 1505-1525 cm^{-1} [4].

Clinical 1 Experiment details

Setup: Collect 400 spectra for each of 25 clinical isolates (5 *E. coli*, 5 *E. faecalis*, 5 *E. faecium*, 5 *P. aeruginosa*, 5 *S. aureus*) derived from patient samples
    Note: We will refer to clinical isolates as patients for simplicity

Pre-train CNN on 30 reference isolates with antibiotic grouping labels
Randomly sample 10 spectra out of 400 for each patient

for fold $\leftarrow 1:5$ **do**
    Assign 1 patient to test set and 4 patients to training set for each species
    Fine-tune CNN on 20 training set patients
    Use fine-tuned CNN to make predictions for all 400 spectra for 5 patients in test set
end for

for trial $\leftarrow 1:10000$ **do**
    Randomly select 10 predictions for each patient
    Diagnose all 25 patients using majority vote
    Record diagnosis accuracy for trial: accuracy $= \frac{\#\text{ correct}}{25}$
end for

Compute average accuracy and standard deviation over all trials

Supplementary Note 1: Pseudocode for fine-tuning and identification of clinical spectra.

Supplementary References

1. Banaei, N., Watz, N., Getsinger, D. & Ghafghaichi, L. SUH antibiogram data for bacterial and yeast isolates. Tech. Rep., Stanford Healthcare Clinical Microbiology Laboratory (2016). URL `http://med.stanford.edu/bugsanddrugs/clinical-microbiology/_jcr_content/main/panel_builder/panel_0/download_748639600/file.res/SHC\%20antibiogram\202016.pdf`.

2. Nakasone, T. *et al.* Bacterial susceptibility report: 2016. Tech. Rep., VA Palo Alto Health Care System (2017). URL `https://web.stanford.edu/~jonc101/tools/Antibiogram/VAPAabgm2016Report\%20FINAL4-14-17.pdf`.

3. Diep, B. A. *et al.* The arginine catabolic mobile element and staphylococcal chromosomal cassette mec linkage: convergence of virulence and resistance in the USA300 clone of methicillin-resistant staphylococcus aureus. *J. Infect. Dis.* **197**, 1523–1530 (2008).

4. Lorenz, B., Wichmann, C., Stöckel, S., Rösch, P. & Popp, J. Cultivation-Free raman spectroscopic investigations of bacteria. *Trends Microbiol.* **25**, 413–424 (2017).
