# Supplementary material for: Rapid identification of pathogenic bacteria using Raman spectroscopy and deep learning
Source: Nat Commun. 2019 Oct 30;10:4927. doi: 10.1038/s41467-019-12898-9 (PMC6960993; doi:10.1038/s41467-019-12898-9)
Supplement: Supplementary file 2 — Reporting Summary [file 41467_2019_12898_MOESM2_ESM.pdf]

nature research

Corresponding author(s): Chi-Sing Ho, Niaz Banaei, Amr Saleh, Stefano Ermon, Jennifer Dionne

Last updated by author(s): Sep 11, 2019

# Reporting Summary

Nature Research wishes to improve the reproducibility of the work that we publish. This form provides structure for consistency and transparency in reporting. For further information on Nature Research policies, see Authors & Referees and the Editorial Policy Checklist.

## Statistics

For all statistical analyses, confirm that the following items are present in the figure legend, table legend, main text, or Methods section.

| n/a | Confirmed | |
|---|---|---|
| ☐ | ☒ | The exact sample size (*n*) for each experimental group/condition, given as a discrete number and unit of measurement |
| ☐ | ☒ | A statement on whether measurements were taken from distinct samples or whether the same sample was measured repeatedly |
| ☒ | ☐ | The statistical test(s) used AND whether they are one- or two-sided<br>*Only common tests should be described solely by name; describe more complex techniques in the Methods section.* |
| ☐ | ☒ | A description of all covariates tested |
| ☒ | ☐ | A description of any assumptions or corrections, such as tests of normality and adjustment for multiple comparisons |
| ☐ | ☒ | A full description of the statistical parameters including central tendency (e.g. means) or other basic estimates (e.g. regression coefficient) AND variation (e.g. standard deviation) or associated estimates of uncertainty (e.g. confidence intervals) |
| ☐ | ☒ | For null hypothesis testing, the test statistic (e.g. *F*, *t*, *r*) with confidence intervals, effect sizes, degrees of freedom and *P* value noted<br>*Give P values as exact values whenever suitable.* |
| ☒ | ☐ | For Bayesian analysis, information on the choice of priors and Markov chain Monte Carlo settings |
| ☒ | ☐ | For hierarchical and complex designs, identification of the appropriate level for tests and full reporting of outcomes |
| ☐ | ☐ | Estimates of effect sizes (e.g. Cohen's *d*, Pearson's *r*), indicating how they were calculated |

*Our web collection on statistics for biologists contains articles on many of the points above.*

## Software and code

Policy information about availability of computer code

| | |
|---|---|
| Data collection | HORIBA Scientific's LabSpec 6 was used to collect the spectral data from the HORIBA Labram spectrometer. |
| Data analysis | Custom python code was used for classification and analysis. This code is published at https://github.com/csho33/bacteria-ID. The Matlab subbackmod function in the publicly available Biotools Matlab toolbox was used for spectral background subtraction. |

For manuscripts utilizing custom algorithms or software that are central to the research but not yet described in published literature, software must be made available to editors/reviewers. We strongly encourage code deposition in a community repository (e.g. GitHub). See the Nature Research guidelines for submitting code & software for further information.

## Data

Policy information about availability of data

All manuscripts must include a data availability statement. This statement should provide the following information, where applicable:

- Accession codes, unique identifiers, or web links for publicly available datasets
- A list of figures that have associated raw data
- A description of any restrictions on data availability

All data and code needed to replicate these results are available at https://github.com/csho33/bacteria-ID. Figure 1d, SI Figure 1 and SI Figure 4 have associated raw data.

# Field-specific reporting

Please select the one below that is the best fit for your research. If you are not sure, read the appropriate sections before making your selection.

☒ Life sciences ☐ Behavioural & social sciences ☐ Ecological, evolutionary & environmental sciences

For a reference copy of the document with all sections, see nature.com/documents/nr-reporting-summary-flat.pdf

# Life sciences study design

All studies must disclose on these points even when the disclosure is negative.

| | |
|---|---|
| Sample size | No sample size calculations were performed. Sample size was determined to be adequate based on the consistency of classification across 5 randomly selected train and validation splits. |
| Data exclusions | For the reference database, we exclude the spectra that are most likely to be non-monolayer measurements by ranking the spectra by signal intensity and discarding the 25 spectra with highest intensity, which includes all spectra with intensities greater than two standard deviations from the mean. Exclusion criteria were pre-established. |
| Replication | All machine learning experiments were replicated successfully across 5 randomly selected train and validation splits. All clinical machine learning experiments were replicated successfully across 10,000 random sampling trials. |
| Randomization | In our clinical experiments, samples were assigned at random while preserving the percentage of samples for each class to train, validation, and test sets through standard cross validation procedures. In our reference experiments, we report results with both possible ordering of samples used for test and fine-tuning. |
| Blinding | The investigators were not blinded during data collection and analysis. Blinding was not relevant to our study due to the inherently blinded nature of test procedures. |

# Reporting for specific materials, systems and methods

We require information from authors about some types of materials, experimental systems and methods used in many studies. Here, indicate whether each material, system or method listed is relevant to your study. If you are not sure if a list item applies to your research, read the appropriate section before selecting a response.

## Materials & experimental systems

| n/a | Involved in the study | |
|---|---|---|
| ☒ | ☐ | Antibodies |
| ☒ | ☐ | Eukaryotic cell lines |
| ☒ | ☐ | Palaeontology |
| ☒ | ☐ | Animals and other organisms |
| ☒ | ☐ | Human research participants |
| ☒ | ☐ | Clinical data |

## Methods

| n/a | Involved in the study | |
|---|---|---|
| ☒ | ☐ | ChIP-seq |
| ☒ | ☐ | Flow cytometry |
| ☒ | ☐ | MRI-based neuroimaging |
